# Oxidized Starch-Reinforced Aqueous Polymer Isocyanate Cured with High-Frequency Heating

**DOI:** 10.3390/polym16111609

**Published:** 2024-06-06

**Authors:** Yanrui Li, Detao Kong, Qinghua Yang, Hao Sun, Yaolong He, Nenghui Zhang, Hongjiu Hu

**Affiliations:** 1 Shanghai Institute of Applied Mathematics and Mechanics, School of Mechanics and Engineering Science, Shanghai University, Shanghai 200072, China; 13356664513@shu.edu.cn (Y.L.); kongdt1996@shu.edu.cn (D.K.); yang_qh@shu.edu.cn (Q.Y.); 21sunhao@shu.edu.cn (H.S.); 2 Shanghai Key Laboratory of Mechanics in Energy Engineering, Shanghai 200072, China; 3 Shanghai Frontier Science Center of Mechanoinformatics, Shanghai 200072, China

**Keywords:** aqueous polymer isocyanate, oxidized starch, high frequency, mechanical properties, bonding performance

## Abstract

In this research, an oxidized starch/styrene–butadiene rubber system with high capability of absorbing electromagnetic energy was adopted as the main component, the effect of oxidized starch content on the bonding and mechanical properties of aqueous polymer isocyanate (API) after high-frequency curing was evaluated, and the effect mechanisms were explored by combining thermodynamic tests and material characterization methods. Our findings revealed that the addition of oxidized starch enhanced the mechanical properties of API after high-frequency curing and the increase in the amount of oxidized starch enhanced the improvement effect of high-frequency curing on API bonding and mechanical properties. At 5 wt% oxidized starch, high-frequency curing improved API bonding properties by 18.0% and 17.3% under ambient conditions and after boiling water aging, respectively. An increase in oxidized starch content to 25 wt% increased enhancement to 25.1% and 26.4% for the above conditions, respectively. The enhancement effects of tensile strength and Young’s modulus of the API adhesive body were increased from 9.4% and 18.2% to 18.7% and 22.6%, respectively. The potential enhancement mechanism could be that oxidized starch could increase the dielectric loss of API, converting more electromagnetic energy into thermal energy creating more cross-linked structures.

## 1. Introduction

Aqueous polymer isocyanate (API) consists of isocyanate and a main component (MC), which generally contains aqueous polymers with active hydrogen, organic/inorganic fillers, and additives [1,2,3]. API has excellent bonding characteristics, a wide range of applications, and no stringent moisture content requirements for bonded wood; therefore, it is extensively employed in load-bearing structures, such as small- and medium-sized glulam beams in interiors [4]. Although API has been extensively applied in a wide range of industries, there are still many problems that need to be solved. Firstly, the cost of the main raw materials in these bonding systems (MC and cross-linking agent) and thermal curing costs are high [5]; secondly, an increase in isocyanate content improves the tensile strength and creep resistance of composite materials, but the excessive addition of isocyanate significantly shortens the API active period [6]. Furthermore, an excessive amount of cross-linker significantly decreases the room temperature bond strength [7], fracture toughness [8], and physical aging resistance [9] of API. However, a decrease in the cross-linker amount seriously weakens the long-term service performance of API in hot and humid environments. Solving the above problems is an important research topic in the field of API.

The service performance of API bonding structures Is closely related to their curing process. Therefore, the curing process is an effective way to solve the current problems of API. Room temperature curing consumes less energy and decreases production costs to a certain extent, but its disadvantage is that it takes relatively long times. The post-curing process can effectively decrease the amount of residual -NCO in API film and enhance the cross-linking degree of polymers to improve API moisture resistance [10], but it takes a long time and consumes high amounts of energy. High-temperature curing shortens the production cycle, but low energy efficiency results in high costs; uneven curing also affects the strength of the bonded structures. Hence, a new curing process is urgently required to solve the above problems. High-frequency curing has become a research hotspot in recent years because of its numerous advantages, including fast heating speed, controllable temperature, uniform heating, high energy efficiency, and low cost [11,12,13], and has a good development prospect in the field of adhesive curing.

Several research works have verified that high-frequency electromagnetic fields could be applied for curing different material-component adhesives. Compared to conventional curing, the high-frequency curing process can provide equivalent or better mechanical characteristics [12,14] and bonding performance of the adhesive with shorter curing times [15]. The experimental results of Horikoshi et al. showed the high-frequency heating effect on components and its role in the chemical reaction process [16]. Zhou et al. found that high-frequency heating significantly increased polymer curing rates [17]. Chiozza et al. studied the high-frequency curing effect on the bonding of wood with polyvinyl acetate dispersion adhesives and showed that, although high-frequency curing did not remarkably enhance adhesive bonding performance, it significantly decreased bonded structure water content and, at the same time, greatly reduced the curing time [18]. Wang et al. studied the influences of high-frequency curing and heat curing on phenylethynyl adhesives and found that the compression shear strength of high-frequency-cured bonded specimens was comparable to ambient temperature-cured ones; however, high-frequency curing significantly decreased curing time and improved energy efficiency [14]. Li et al. also investigated the high-frequency hot-pressing mechanism and gluing characteristics of soybean glue plywood and showed that, during the process of high-frequency hot pressing, plywood internal temperature field distribution was very uniform [19]. Park et al. studied the shear and flexural characteristics of bonded laminated wood after high-frequency heating and found that high-frequency curing promoted the process of liquid vinyl acetate adhesive penetration into the wood, which increased the adhesive shear strength and bonded structure bending [20]. Olofinjana et al. explored a variety of factors affecting bonding performance under high-frequency curing conditions, such as temperature control, adhesive type, and bonding surface treatment [21].

Starch contains a great number of reactive groups, has a relatively high dielectric loss, and is also the cheapest carbohydrate in the industry [22]. In this research, the oxidized starch/styrene–butadiene rubber (SBR) system, which has strong electromagnetic energy absorption, was selected as the MC to be mixed with isocyanate to prepare API. High-frequency curing treatment was performed on the adhesive structure and API body to evaluate the effect of oxidized starch content on API high-frequency curing performance. Firstly, we performed tensile and compression shear tests on API adhesives under room temperature and boiling water aging, respectively. Then, dielectric testing and differential scanning calorimetry (DSC) were performed to evaluate the dielectric loss and specific heat capacity of API adhesives. In addition, Fourier transform infrared (FTIR) spectral analysis and scanning electron microscopy (SEM) were applied to evaluate the microstructure characteristics of API adhesives, aiming to investigate the potential effect mechanism of starch content on API mechanical properties after high-frequency curing.

## 2. Materials and Methods

### 2.1. Preparation of API

The MC mainly consisted of oxidized starch, hydroxyethyl cellulose (HEC, Aladdin Biochemical Technology Co., Ltd., Shanghai, China), sodium tetraborate (Aladdin Biochemical Technology Co., Ltd., Shanghai, China) mixed solution, styrene butadiene rubber emulsion (SBR, Trinseo S.A. Shanghai Branch, Shanghai, China), and calcium carbonate (CaCO_3_, 800 mesh, Sinopharm Chemical Reagent Co., Ltd., Shanghai, China). The mass concentrations of oxidized starch in mixed solutions were 5, 10, 15, 20, and 25 wt%, the concentration of HEC was 4%, and the concentration of sodium tetraborate was 1%. SBR accounted for one third of the mass of the main agent and CaCO_3_ content was adjusted according to the content of oxidized starch, so that the CaCO_3_ and oxidized starch accounted for one third of the mass of the main agent. Also, we adopted standard industrial p-MDI (Rubinate 5005, Huntsman Polyurethane Co., Ltd., Shanghai, China) as the API cross-linker. Table 1 summarizes the properties of the above raw materials in API adhesives. Firstly, various concentrations of oxidized starch were added to deionized water and stirred for 70 min at 70 °C. After cooling the solution to 30 °C, HEC and sodium tetraborate were added and stirred for 1 h, then SBR was added and stirred for a further 90 min at 60 °C. The above stirring operation was achieved by an electric stirrer (LC-ES-60SH, Shanghai Lichen Instrument Technology Co., Ltd., Shanghai, China) with a controlled speed of 400 rpm, and temperature control was achieved by a thermostatic bath (CH1006, Haorui Experimental Instrument Co., Ltd., Yancheng, China), where the flask containing the solution was placed in a thermostatic bath in order to control the warming or cooling of the solution. Then, the solution was cooled down to room temperature and transferred to a planetary stirrer (PBM-M-0.4A, Shenzhen Jitong Technology Development Co., Ltd., Shenzhen, China), and the MC was obtained by adding CaCO_3_ particles and stirring at 600 rpm to mix it completely. API adhesive was obtained by mixing an isocyanate cross-linking agent with the above MC with a mass ratio of 15:100.

### 2.2. Sample Preparation

**Figure 1:**
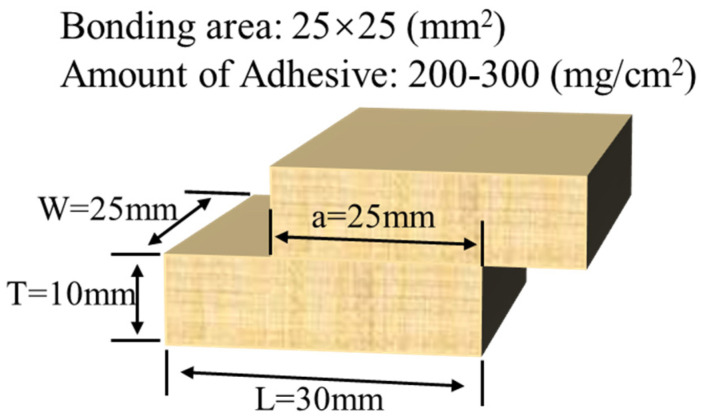
Lap joint specimen of wood.

**Figure 2:**
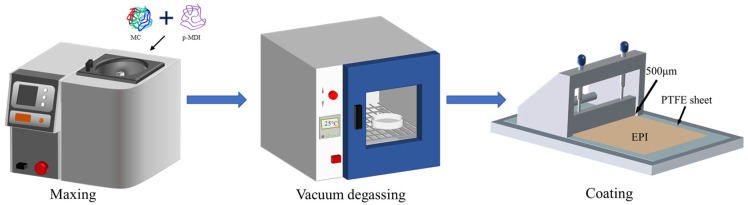
Flowchart of API film preparation.

(1) API-bonded wood specimens: Rubberwood (Brazilian rubber tree) with 8.0 ± 2.0% moisture content was cut into smooth-surfaced blocks with dimensions of 30.0 mm (L) × 25.0 mm (W) × 10.0 mm (T) and a bonding area of 25.0 × 25.0 mm^2^; API amount was 200–300 (mg/cm^2^); and fiber direction was parallel to axial loading direction. Lap-cut specimens were prepared according to the technical guideline of JIS K6806, as illustrated in Figure 1. Then, bonded specimens were cured in a high-frequency microwave generator (2.45 GHz, 700 kw, Midea Group Ltd., Foshan, China) at 1 MPa for 10 min (the time required for the bonded specimens to be fully cured with minimum oxidized starch content). At the same time, specimens were prepared in accordance with the ambient temperature curing procedure of JIS K6806 standard [24] (30% relative humidity, ambient temperature curing at 10 MPa for 24 h, then remove the pressure and continue curing for 72 h) to compare them with specimens after high-frequency curing.

(2) Curing API films: Figure 2 presents the API adhesive film preparation process. Firstly, in order to mix the main agent with the cross-linker, the two were mechanically mixed in a planetary stirrer at 600 rpm for 10 min, and then the mixed emulsion was degassed in a vacuum oven (DZF-6020, Shanghai Jiecheng Experimental Instrument Co., Shanghai, China) at ambient temperature for 5 min. Then, using an automatic squeegee coater (MS-ZN320B, Xiamen Maosen Automation Equipment Co., Xiamen, China), the mixture was coated on a tetrafluoro plate and part of the API film was put into the high-frequency generator to cure for 10 min; part of the film was cured at 25 °C at 30% relative humidity for 72 h. The dried film with 140 μm thickness was stored as a sample in an argon-protected glove box to test after curing. After curing, another dried film sample with 140 μm thickness was stored in an argon-protected glove box for testing.

### 2.3. Characterization of Oxidized Starch-Modified API Adhesive

#### 2.3.1. Fourier Transform Infrared (FTIR) Spectroscopy

In order to identify chemical group variations in API adhesives with different contents of oxidized starch after high-frequency curing with ambient temperature curing, and to reveal underlying chemical mechanisms, FTIR was used to measure the API film with reference to ISO 20368:2017 [25].

FTIR spectra were measured with an Avatar Nicolet 380 spectrometer (Thermo Fisher Scientific, Waltham, MA, USA) using the KBr tablet method. A total of 16 total reflectance infrared spectral scans were recorded on each cured API film using a Thermo Scientific Nicolet iS50 with an intelligent iTR diamond ATR crystal. Wavelength was set in the range of 650~4000 cm^−1^ and IR beam average penetration depth was 2.03 μm.

#### 2.3.2. EIS

The electromagnetic radiation absorption capacity of a material per unit volume can be calculated as [26,27,28]:

(1) $$P=2\pi f{E_{1}}^{2}\varepsilon^{\prime \prime }$$

where *P* is absorbed energy per unit volume (W/m^3^), *f* is frequency (Hz), *E*_1_ is electric field strength (V/m), and *ε*″ is dielectric loss. From Equation (1), it is seen that at certain values of electric field strength and frequency, the increase in dielectric loss, *ε*″, enhances the material’s ability to convert electromagnetic energy into thermal energy.

The alternating current (AC) impedance value of the API emulsion in an alternating electric field was measured by an electrochemical workstation (CHI660E, Zahner, German), and there was the following relationship between *ε*″ and AC impedance of the material [29,30]:

(2) $$\varepsilon ''=\frac{Z'}{2\pi fC_{0}(Z{'}^{2}+Z'{'}^{2})}$$

Where *Z*′ and *Z*″ are complex impedance values measured by the AC impedance method and *C*_0_ is the vacuum capacitance (F/m).

Therefore, API dielectric loss was measured using an electrochemical workstation. API emulsions with different contents of oxidized starch were loaded into a model cell and API emulsion ionic conductivity was measured using a CHI660E electrochemical workstation. In the test, open circuit voltage was 0 V, perturbation voltage was 5 mV, and frequency range was 0.01–100,000 Hz to obtain AC impedance diagrams of different API emulsions. Then, API dielectric loss was calculated by Equation (2).

#### 2.3.3. DSC

In this research, API emulsion specific heat capacity was determined by the indirect method using DSC Q2000 (TA Instrument@, TA Instruments-Waters LLC, New Castle, DE, USA). Since the variations in specific heat capacity with temperature were very small and the effect of API phase change had to be excluded, we only used API specific heat capacity in the range of 25–60 °C to represent API specific heat capacity during the whole high-frequency curing process. The specific heat capacities of the samples were obtained by holding the samples at 25 °C for 10 min, increasing the temperature at a 5 °C/min rate to 60 °C, and then holding the samples at this temperature for another 10 min. The heat flow curves of baseline, sapphire standard, and samples were drawn using the same procedure, and the specific heat capacities of the samples were calculated by Equation (3).

(3) $${c_{p}}^{sp}={c_{p}}^{\mathrm{cal}}\cdot \frac{m^{\mathrm{cal}}(P_{\mathrm{sp}}-P_{\mathrm{blank}})}{m^{\mathrm{sp}}(P_{\mathrm{cal}}-P_{\mathrm{blank}})}$$

where *m* is mass (mg), *c*_p_ is specific heat capacity (J·g^−1^·°C^−1^), *P* is heat flow (mW), superscripts sp and cal represent the API sample and specimen, respectively, and *P*_blank_ denotes baseline heat flow.

Assuming that the dielectric loss and specific heat capacity of API were known, the following equation was applied to calculate the rate of temperature increase per API unit mass during high-frequency curing:

(4) $$\frac{\mathrm{dT}}{\mathrm{dt}}=\frac{2\pi f{E_{1}}^{2}\varepsilon "}{\rho \cdot c_{p}}$$

where *ρ* is API emulsion density (g/cm^3^). 

#### 2.3.4. Bulk Tensile Properties

Understanding the deformation and fracture behaviors of adhesive films under mechanical loading is essential for the design and application of API-bonded structures. Hence, it is necessary to perform quasi-static tensile tests on API propriety films.

The uniaxial tensile tests were conducted based on ASTM D882; quasi-static tensile tests were performed on cured API films at 25 °C, 50%RH using a DMA (TA Q800, TA Instruments-Waters LLC, New Castle, DE, USA). The films had dimensions of 40.0 mm (L) × 5.0 mm (W) × 0.14 mm (T) and a strain rate of 5 × 10^−3^/s. Five uniaxial tension runs were performed for each API film with each oxidized starch content, and the averages of the corresponding maximum stress, Young’s modulus, and fracture elongation were determined for each.

#### 2.3.5. SEM Image

In order to explore the effect mechanism of starch content on the adhesive and mechanical properties of API after high-frequency curing, and to investigate the microstructure of API film, the fracture morphologies of API films cured at ambient temperature and after high-frequency curing were observed by SEM (Gemini SEM-300, Carl Zeiss, Jena, Germany).

#### 2.3.6. Compound Characteristics

An important potential application of API as a structural wood adhesive is the production of building components, such as cross-laminated timber and glued laminated timber beams [30]. Hence, two different bond strength tests at ambient temperature and under hygroscopic conditions were needed. For hygroscopic tests, the most accurate method was long-term mechanical tests on API-bonded wooden structures in outdoor environments. However, this was very time consuming and labor intensive. Based on the time–temperature equivalence principle, JIS K6806 advised the following accelerated test methods for API:

(1) Submerging API-bonded woods in boiling water for 4 h and drying by hot air at 60 ± 3 °C for 20 h.
(2) Repeating the above process one more time.
(3) The specimens were cooled in water at room temperature and loaded at a 9.0 kN/min compression speed.

Based on the above experimental procedure, we performed compression tests on API-bonded wood lap specimens using a universal testing machine (Zwick-Z020, Jinan Dongfang Experimental Instrument Co., Jinan, China) and tested 12 API-bonded wood lap specimens for each oxidized starch content according to the test requirements of JIS K6806, and then took the average value as the bond strength.

## 3. Results

### 3.1. Chemical Groups of Cured API Film

In order to gain a more comprehensive understanding of the oxidized starch strengthening effect, it is necessary to investigate variations in thermodynamic properties, chemical groups, and API microstructure after ambient temperature and high-frequency curing with different oxidized starch contents. Therefore, we discuss the strengthening effect of oxidized starch on API after high-frequency curing in terms of chemical and physical effects in the following section.

**Figure 3:**
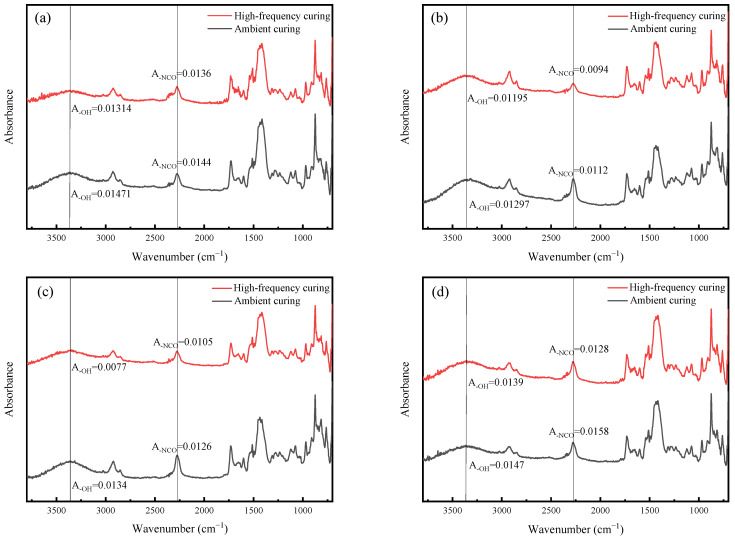
Infrared spectra of API at different oxidized starch contents of (**a**) 5 wt%, (**b**) 10 wt%, (**c**) 15 wt%, and (**d**) 20 wt%.

The internal reactions of API adhesives are extraordinarily complex, and the published consensus is that the isocyanate group (-NCO) reacts primarily with hydroxyl groups and other reactive functional groups (e.g., carboxyl groups and amines) to form solid cross-linking structures, such as urethanes amide and biuret. To explore the chemical mechanism of API enhancement by oxidized starch after high-frequency curing, we performed FTIR spectroscopy on API films. The experimental results demonstrate the mechanical properties of API at 20 wt% oxidized starch contents are similar to 25%. Therefore, we performed infrared testing on API after three days of ambient temperature curing and 10 min high-frequency curing in oxidized starch content in the range of 5–20 wt%, and the obtained results are illustrated in Figure 3.

As can be seen in the figure, the intensities of the -NCO absorption peak at 2270 cm^−1^ (A_NCO_) and -OH at 3360 cm^−1^ (A_OH_) were decreased after the high-frequency curing of API for all starch oxide contents. This indicated that the -NCO group had undergone a more complete cross-linking reaction with the -OH group, which improved the mechanical stability of the API adhesive structure. In addition, it was found that the reduction in -NCO groups was increased with the increase in oxidized starch content. The reduction in -NCO groups increased from 5.6 to 19.0% when the oxidized starch content increased from 5 to 20 wt%. The evolution of A_NCO_ corroborated that high-frequency curing promoted the reaction of isocyanate and reactive functional groups in API to build a solid cross-linked structure, and that the enhancement magnitude of the adhesive and mechanical properties of API following high-frequency curing were increased with the increase in starch content.

### 3.2. Dielectric Properties and Specific Heat Capacity of the API Adhesive

**Figure 4:**
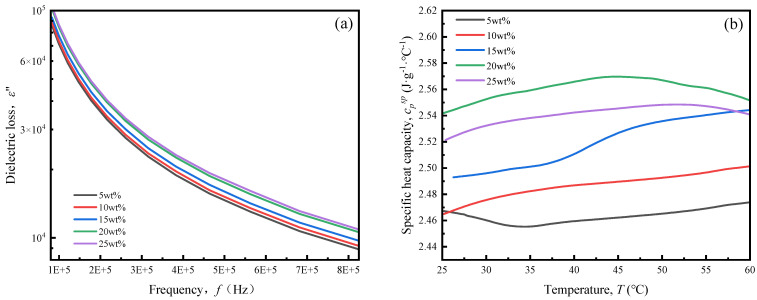
(**a**) Dielectric loss and (**b**) specific heat capacity of API samples with different contents of oxidized starch.

The dielectric loss and specific heat capacity variation curves of API samples with different contents of oxidized starch are illustrated in Figure 4a,b.

According to Equation (3), the API absorption capacity for electromagnetic energy during high-frequency curing mainly depends on dielectric loss. From Figure 4a, it can be seen that dielectric loss gradually increases with the increase in the content of oxidized starch. The increase in oxidized starch content from 5 to 25 wt% enhances its dielectric loss by 21.1% at a 82,500 Hz frequency. The main reason for this is the presence of a large number of polar groups in the oxidized starch, which constantly move and rub against each other to generate heat after applying a high-frequency alternating electric field [31], increasing API dielectric loss. This facilitates the generation of more heat during the curing process and accelerates a molecular thermal movement-promoted cross-linking reaction to produce more cross-linked structures. 

Figure 4b illustrates that the API emulsion specific heat capacity does not change much by changing the oxidized starch content. It is also found that the difference between the highest and lowest values is only 4.2% and there is no obvious linear relationship with the oxidized starch content. This might be due to the fact that the specific heat capacities of oxidized starch and CaCO_3_ are close to each other and both are much smaller than those of water, so that the variation in API specific heat capacity is not significant when the total contents of both components remain constant. The density of API emulsions with different oxidized starch contents are presented in Appendix A. It is witnessed that an increase in the content of oxidized starch to some extent decreases API emulsion density. This may be due to oxidized starch substituting for denser CaCO_3_.

Overall, the above analyses show that oxidized starch could improve API dielectric loss properties and decrease API density, but have a slight effect on specific heat capacity. According to Equation (4), the addition of oxidized starch increases the API emulsion heating rate during high-frequency curing, which can accelerate the occurrence of cross-linking reactions and API emulsion penetration into the interior of the wood, so that adhesive strength enhancement by high-frequency curing is more obvious by the increase in starch content.

**Figure 5:**
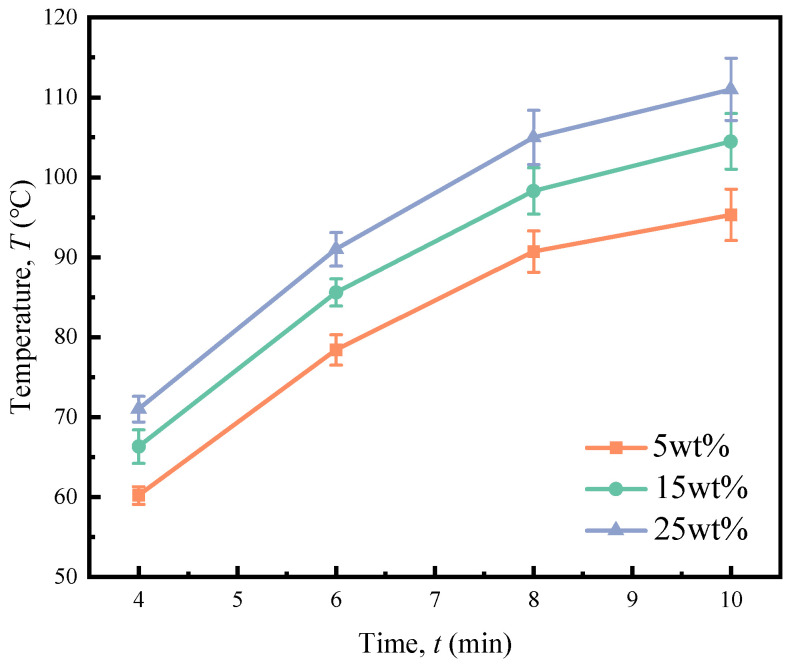
Temperature variations in API containing various amounts of starch during the high-frequency curing process.

The temperature variations in API adhesives with different starch contents during high-frequency curing are shown in Figure 5. During the initial 8 min of high-frequency curing, the temperature rises rapidly. However, between 8 and 10 min, the temperature increases slowly due to approaching thermal equilibrium [20]. Meanwhile, we found that API temperature during high-frequency curing increases by increasing the oxidized starch content. By increasing the oxidized starch content from 5 to 25 wt%, the temperature after 10 min of high-frequency curing rises from 95 °C to 111 °C. This observation aligns with the predictions made using Equation (4).

### 3.3. Quasi-Static Tensile Behavior of Cured API Films

**Figure 6:**
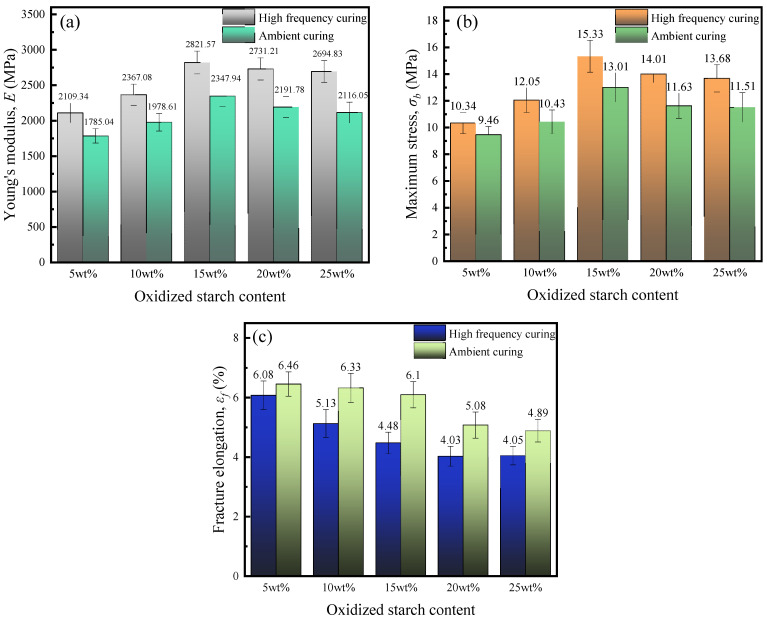
Mechanical properties of API bodies with different oxidized starch contents: (**a**) Young’s modulus, (**b**) maximum stress, and (**c**) fracture elongation.

We performed quasi-static tensile experiments on an API body and obtained its Young’s modulus *E*, maximum stress (*σ*_b_), and fracture elongation (*ε*_f_), as illustrated in Figure 6.

From Figure 6a,b, it can be seen that Young’s modulus and maximum stress of API films present a trend of rapid increase followed by slow decrease by increasing the starch content. The main reason for this is that, as the oxidized starch content increased, more reactive groups, such as hydroxyl groups, reacted with isocyanate, improving API body quasi-static tensile properties. However, when the oxidized starch content is too high, oxidized starch particles in the solution are prone to agglomeration, which not only make API films’ performance non-uniform, but also block the cross-linking structure generated by the reaction with isocyanate, resulting in decreases in Young’s modulus and maximum stress. From Figure 6c, it is seen that API fracture elongation decreases with the increase in oxidized starch content, but the magnitude of the decrease is not significant, with a starch content range of 5~15 wt%, but becomes more significant when the starch content is 20~25 wt%. This might be due to the uneven dispersion of API components due to too-high oxidized starch contents, resulting in a rapid decrease in fracture elongation.

The high-frequency curing effect intensity on the API body’s mechanical properties increases with the increase in oxidized starch content. At 5 wt% oxidized starch content, Young’s modulus and maximum stress of the API body after high-frequency curing are enhanced by 18.2% and 9.4%, respectively, compared to ambient temperature curing. When the oxidized starch content reaches 25 wt%, enhancement degrees are increased to 22.6 and 18.7%. This might be due to the ability of oxidized starch to improve electromagnetic energy absorption in the API body and the increase in starch content promoted the reaction of various groups, such as hydroxyl with isocyanate, to generate more cross-linked structures.

**Figure 7:**
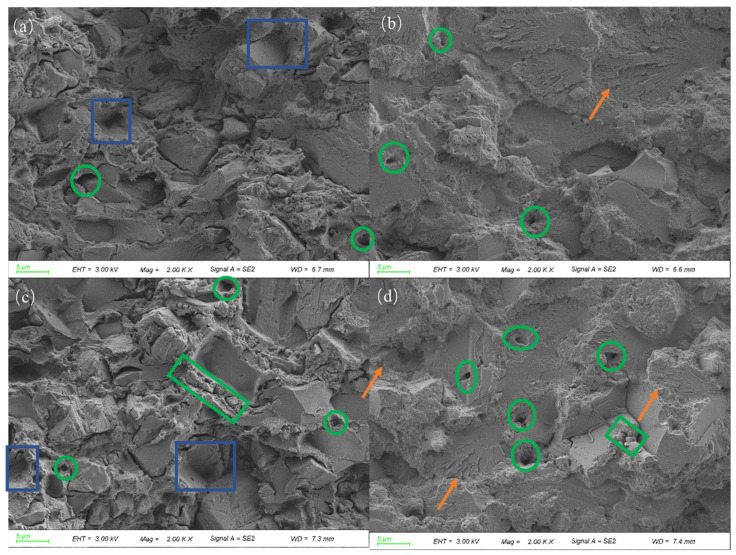
SEM images of API fracture (blue boxes: pits, green cycles: pores, green boxes: agglomeration of starch, orange arrow: microcracks): (**a**) 15 wt% starch content and high-frequency-cured, (**b**) 15 wt% starch content and ambient temperature-cured, (**c**) 20 wt% starch content and high-frequency-cured, and (**d**) 20 wt% starch content and ambient temperature-cured.

In order to investigate the effect mechanism of starch content on API mechanical property enhancement after high-frequency curing, we selected two API fracture types in samples containing 15 and 20 wt% oxidized starch for SEM analysis, and the results are illustrated in Figure 7.

Following high-frequency curing, Figure 7a,c show that API fracture becomes rougher and air bubbles disappear, creating several pits at fracture (blue boxes), which indicates that material body interfacial properties have significantly improved. Therefore, API has a higher fracture resistance after high-frequency curing [32]. From the orange arrows in Figure 7b,d, it can be seen that a large number of microcracks exist in the API fracture after ambient curing, which weaken the mechanical properties of the API adhesive. High-frequency curing enhances the effect of starch content on API bonding properties. This is primarily because electromagnetic radiation accelerates water evaporation and the cross-linking reaction, reducing residual -NCO groups and subsequently decreasing their reaction with water and other active substances to produce CO_2_.

When the oxidized starch content reached 20 wt%, the agglomeration of starch granules had already occurred (green box), which was not conducive to the cross-linking reaction. Reduced cross-linking reactions result in more residual -NCO groups in API. These -NCO groups react with substances containing active groups, such as water, generating a large amount of gas and increasing the internal pores (green cycles) in API adhesives. This conclusion can also be drawn by comparing the API SEM images with 15% and 20% starch contents. Therefore, a further increase in the oxidized starch content will weaken the bond strength of the components rather than increasing it.

### 3.4. Bonding Performance of the API Adhesive

**Figure 8:**
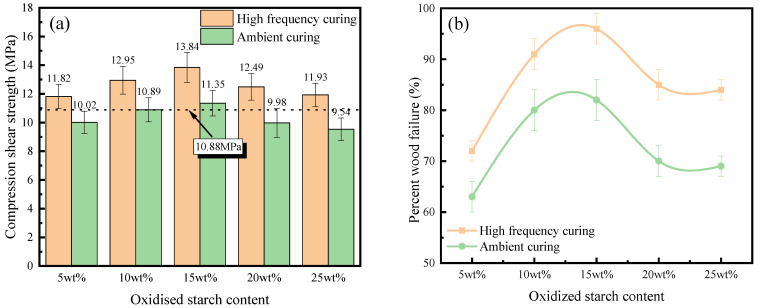
(**a**) Compressive shear strength and (**b**) percent wood failure of API-bonded specimens containing different amounts of oxidized starch (room temperature).

**Figure 9:**
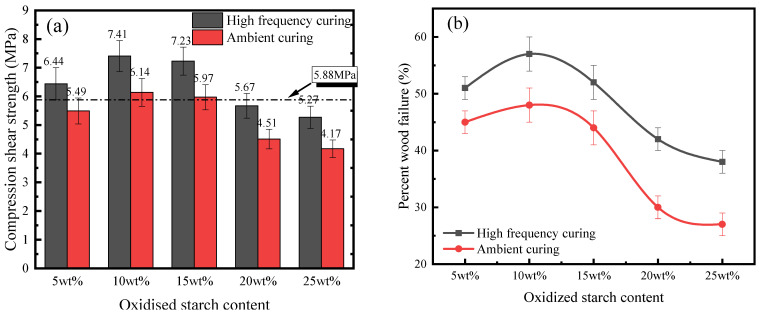
(**a**) Compressive shear strength and (**b**) percent wood failure of API-bonded specimens containing different amounts of oxidized starch (boiling water aging).

Based on JIS K6806, Figure 8 shows the obtained ultimate stress and percent wood failure of API-glued wood specimens subjected to compressive shear loading to mechanical damage via room temperature tests. API-glued wood specimens were subjected to boiling water, cool water, and hot air before compressive shear to mechanical failure. The obtained ultimate stress and percent wood failure are shown in Figure 9.

As is seen from Figure 8a and Figure 9a, the bonding performance of the bonded specimens after high-frequency curing is remarkably improved under both ambient conditions and after boiling water aging. This is because of high-frequency microwaves, since they rapidly and uniformly increase the internal temperature of the adhesive layer [19] and molecular movement is accelerated to promote high-strength-polymer mesh-structure formation, thus improving the ability of the adhesive layer to resist mechanical damage. In addition, high-frequency microwaves promote API penetration into the wood interior [20], where they react with hydroxyl and other groups to enhance the interfacial properties of bonded components, so that the position of the structure hazard is shifted to the wood body, as witnessed by the improvement of percent wood failure in Figure 8b and Figure 9b. It is also seen that bond strength enhancement by high-frequency curing increases as the content of oxidized starch increases. At a 5 wt% oxidized starch content, the compression shear strength of boiling water aging and room temperature conditions after high-frequency curing are enhanced by 18.0% and 17.3% respectively, compared to those obtained by ambient curing; enhancement effects are increased to 25.1% and 26.3%, respectively, when the content of oxidized starch reaches 25 wt%. The main reason is that oxidized starch promotes electromagnetic energy absorption by API, helping to heat up the interior of API-glued wood specimens.

At room temperature, the bonding properties of API adhesives with all oxidized starch contents following high-frequency curing meet the requirements of the JIS K6806 standard (10.88 MPa at room temperature and 5.88 MPa after aging). After aging, the bonding performance of samples containing starch contents higher than 15 wt% is lower than that required by JIS K6806 (No. 1-1). This might be due to the poor boiling water resistance of oxidized starch [33,34], and a too-high oxidized starch content cannot improve bonding properties after boiling water aging.

API-glued wood specimen bonding properties after high-frequency curing, both at room temperature conditions and after aging, present a trend of first increasing and then decreasing by increasing the content of oxidized starch. This might be due to the fact that, with the increase in oxidized starch content, there are more active groups, such as hydroxyl groups, in the material and the reaction with the cross-linking agent is more complete. However, when the oxidized starch content is too high, API viscosity is also too high (25.9 Pa.s for 15 wt% and 67.8 Pa.s for 25 wt% oxidized starch), which leads to poor fluidity, which is not conducive to API penetration into the interior of the wood and weakens the interfacial properties. On the other hand, if the oxidized starch content is too high, it could easily agglomerate and is difficult to completely disperse, and hydroxyl groups could not be released to a large extent, inhibiting their sufficient reaction with isocyanate, decreasing the bonding performance.

## 4. Conclusions

As an energy-efficient curing method, high-frequency curing has been extensively applied in the adhesive curing process. In this research, the effects of oxidized starch content on the bonding and intrinsic mechanical properties of API following high-frequency curing were studied, and the underlying physical and chemical mechanisms were revealed. The main conclusions of this work were as follows:

(1) The dielectric loss of API was significantly increased by oxidized starch, which improved the ability of API for converting electromagnetic energy into thermal energy, remarkably promoting reactions among isocyanate, MC hydroxyl, and other reactive groups resulting in the formation of more solid cross-linking structures. At the same time, it reduced the residue of the -NCO group to decrease the defects in the API body.
(2) The bonding and mechanical properties of API following high-frequency curing presented a trend of first increasing and then decreasing with the increase in oxidized starch content. When the oxidized starch content was 15 wt%, the optimum bonding and mechanical properties of API were obtained at room temperature, while the bonding property after boiling water aging was at a maximum 10 wt% oxidized starch content.
(3) An increase in the oxidized starch content could promote the enhancement effect of high-frequency curing on the bonding properties of API. The enhancement of bonding properties at room temperature and after boiling water aging was increased from 18 and 17.3% to 25.1 and 26.3% with the increase in the oxidized starch content from 5 to 25 wt%, respectively.
(4) Compared with ambient curing, the Young’s modulus and tensile strength of the API body after high-frequency curing were significantly improved, but its fracture elongation was decreased. Therefore, it could be concluded that the increase in the oxidized starch content resulted in more a pronounced difference between high-frequency curing and ambient curing.

## Figures and Tables

**Table 1:** Characteristics of raw materials in API adhesive.

Main component (MC)	Oxidized starch	Standard	HG/T 3933-2007 [23]
	HEC	Viscosity (mPa.s)	1500
	sodium tetraborate	Fineness	≥99%
	CaCO_3_	Size of particles (um)	15
		Solid content (%)	51
	SBR	pH	6.5
		Viscosity (mPa.s)	101
Cross-linker	p-MDI (Rubinate 5005)	Solid content (%)	100
		NCO (%)	30.5–32.5
		Functionality	2.6–2.7

**Figure A1:**
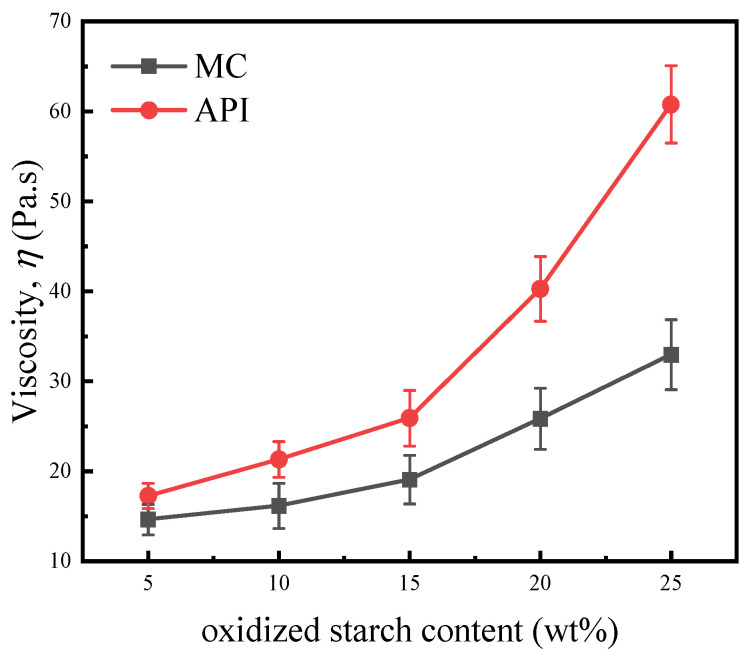
Effect of oxidized starch content on the viscosity of the main agent and API.

## Data Availability

Data are contained within the article.

## Appendix A

**Table A1:** API density with different oxidized starch contents.

Oxidized starch content (wt%)	5	10	15	20	25
Density (g/cm^3^)	1.3	1.28	1.24	1.20	1.17
